# The role of transforming growth factor-beta (TGF-beta) during ovarian follicular development in sheep

**DOI:** 10.1186/1477-7827-2-78

**Published:** 2004-11-25

**Authors:** Jennifer L Juengel, Adrian H Bibby, Karen L Reader, Stan Lun, Laurel D Quirke, Lisa J Haydon, Kenneth P McNatty

**Affiliations:** 1 AgResearch, Wallaceville Animal Research Centre, Upper Hutt, New Zealand

## Abstract

**Background:**

Recently, several members of the transforming growth factor-beta (TGF-beta) superfamily have been shown to be essential for regulating the growth and differentiation of ovarian follicles and thus fertility.

**Methods:**

Ovaries of neonatal and adult sheep were examined for expression of the TGF-betas 1–3 and their receptors (RI and RII) by in situ hybridization using ovine cDNAs. The effects of TGF-beta 1 and 2 on proliferation and differentiation of ovine granulosa cells in vitro were also studied.

**Results:**

The expression patterns of TGF-beta 1 and 2 were similar in that both mRNAs were first observed in thecal cells of type 3 (small pre-antral) follicles. Expression of both mRNAs continued to be observed in the theca of larger follicles and was also present in cells within the stroma and associated with the vascular system of the ovary. There was no evidence for expression in granulosa cells or oocytes. Expression of TGF-beta 3 mRNA was limited to cells associated with the vascular system within the ovary. TGFbetaRI mRNA was observed in oocytes from the type 1 (primordial) to type 5 (antral) stages of follicular growth and granulosa and thecal cells expressed this mRNA at the type 3 (small pre-antral) and subsequent stages of development. The TGFbetaRI signal was also observed in the ovarian stroma and vascular cells. In ovarian follicles, mRNA encoding TGFbetaRII was restricted to thecal cells of type 3 (small pre-antral) and larger follicles. In addition, expression was also observed in some cells of the surface epithelium and in some stromal cells. In granulosa cells cultured for 6 days, both TGF-beta 1 and 2 decreased, in a dose dependent manner, both the amount of DNA and concentration of progesterone.

**Conclusion:**

In summary, mRNA encoding both TGF-beta 1 and 2 were synthesized by ovarian theca, stroma and cells of the vascular system whereas TGF-beta 3 mRNA was synthesized by vascular cells. Luteinizing granulosa cells also responded to both TGF-beta 1 and beta 2 in vitro. These findings in sheep are consistent with TGF-beta potentially being an important autocrine regulator of thecal cell function and possibly a paracrine regulator of ovarian cell function at various development stages.

## Background

Members of the transforming growth factor-beta (TGF-β) superfamily are important intraovarian growth factors [1-6]. Three key members of the TGF-β subfamily, namely TGF-β1, TGF-β2 and TGF-β3, have been shown to be produced by ovarian cells [7-13]. However, the cellular distribution of these proteins varies between species. Likewise, the effects of TGF-βs on granulosa cell function also vary between species. In rodents, TGF-βs are potent stimulators of granulosa cell proliferation [14-16] whereas in other species, such as cattle and pigs, these growth factors have only a mild stimulatory or even inhibitory effect [17-20]. Likewise, TGF-βs stimulate progesterone synthesis from rodent granulosa cells [21-23] where inhibitory effects are observed in granulosa cells collected from sheep, cattle and pigs [17,24-26]. Exceptional ovulation rates and sterility have been observed in lines of sheep with mutations in two members of the TGF-β superfamily, namely growth differentiation factor 9 or bone morphogenetic protein 15 or one of their receptors, activin like kinase-6 [6,27]. However, little is known about the roles of other members of the TGF-β superfamily in sheep and thus the potential interactions of members of the TGF-β superfamily are unclear. The objectives of this study in sheep were to localize the ovarian cellular types expressing mRNA encoding TGF-β1, TGF-β2, TGF-β3, TGFβRI and TGFβRII and to determine the effects of TGF-β1 and TGF-β2 on granulosa cell proliferation/survival and progesterone production *in vitro*.

## Methods

### Generation of cDNAs encoding a portion of the coding region of genes of interest

Except where indicated, laboratory chemicals were obtained from BDH Chemicals New Zealand Ltd (Palmerston North, New Zealand), Invitrogen (Auckland, New Zealand) or Roche Diagnostics N.Z. Ltd. (Auckland, New Zealand).

Total cellular RNA was isolated from ovine ovary using TRIzol according to manufacturer's instructions. First strand cDNA was produced from total cellular RNA using a poly t primer. Complementary DNAs encoding a portion of the coding sequence of the genes were isolated using standard PCR techniques. For individual cDNAs generated, primer sequences and annealing temperature are given in table 1. Resulting PCR products were ligated into appropriate vectors and their nucleotide sequence determined by automated sequence analysis (Waikato DNA Sequencing Facility; The University of Waikato; Hamilton, New Zealand). These sequences were compared with known sequences to confirm identity using the GAP program of GCG (Wisconsin Package Version 10.2, Genetics Computer Group; Madison, Wisconsin). All sequences were >80% identical to those listed as reference sequences in table 1 indicating that the ovine homologue of the respective genes had been obtained.

**Table 1:** GenBank reference numbers used for primer design, primer sequences, annealing temperatures, and GenBank accession numbers for the resulting ovine sequence for the various genes amplified.

Gene	Reference: (Genbank #)	Forward Primer (5' to 3')	Reverse Primer (5' to 3')	Annealing temperature	Genbank # (resulting sequence)
TGF-β1	NM_011577	ggaattcatgccgccctcggggctgcgg (EcoR I site and bases 867–888)	ggtctagatcagctgcacttgcaggagcg (Xba I site and bases 2040–2020)	63°C	ND
TGF-β2	M19154	ggaattcatgcactactgtgtgctgagc (EcoR I site and bases 468–488)	ggtctagagctgcatttrcaagacttkac (Xba I site and bases 1794–1773)	64°C	AY656797
TGF-β3	J03241	ggaattcgcaaagggctctggtggtcctgg (EcoR I site and bases 277–299)	ggtctagaccagttctcctccaagttgcgg (Xba I site and bases 1206–1186)	62°C	AY656798
TGFβRI	U97485	cacagatgggctttgctttg (bases 180–199)	ccttgggtaccaactatctc (bases 1007–988)	50°C	AY656799
TGFβRII	S69114	gtcctgtggacgcgcat (bases 80–97)	aggagcacatgaagaaagtc (bases 449–430)*	50°C	AY656800
TGFβRII (for PCR)	various	gccaacaacatcaaccac	gggtcrtggtcccagca	53°C	AY751461
TGFβRII (internal for PCR)	AY751461	tcgccgaggtctacaagg	atgccctggtggttgagc	55°C	N/A

* Sequence is based on the corresponding ovine sequence obtained from an ovine est clone.

ND, the complete sequence of the clone was not determined, as the ovine TGF-β1 sequence is known. The clone was sequenced from both ends and resulting sequence compared to the known ovine TGF-β1 sequence to confirm identity of the isolated cDNA.

N/A as the sequence overlaps that of AY751461.

### Collection of tissue samples

All experiments were performed in accordance with the 1999 Animal Welfare Act Regulations of New Zealand. All animals had *ad lib *access to pasture and water and lambs were kept with their mothers until just prior to tissue collection. Romney ewes and lambs were killed by administration of a barbiturate overdose (Pentobarbitone; 200 mg/kg, Southern Veterinary Supplies, Christchurch, New Zealand). Recovered ovaries were fixed in 4% (w/v) phosphate-buffered paraformaldehyde and embedded in paraffin wax.

### *In Situ *Hybridization

Cellular localization of mRNAs was determined using the *in situ *hybridization protocol described previously with minor modifications [28]. Sense and anti-sense RNA probes were generated from cDNA encoding the gene of interest with T7, T3 or SP6 RNA polymerase using the Riboprobe combination system (Promega, Dade Behring Diagnostics Ltd., Auckland, New Zealand). For all *in situ *hybridizations, 4–6 μm tissue sections were incubated overnight at 55°C with 45,000 cpm/μl (approximately 48,000 dpm/μl) of ^33^P-labelled antisense RNA. Non-specific hybridization of RNA was removed by RNase A digestion followed by stringent washes (2 × SSC, 50% formamide, 65°C and 0.2 × SSC at 37°C). Following washing, sections were dehydrated, air dried and coated with autoradiographic emulsion (LM-1 emulsion; Amersham Pharmacia Biotech, New Zealand). Emulsion-coated slides were exposed at 4°C for 3 weeks, developed for 3 1/2 minutes in D19 developer (Eastman Kodak, Rochester, NY), development was stopped using a 1 minute incubation in 1% acetic acid and slides were fixed with a 10 minute incubation in Ilfofix II (Ilford Limited, Cheshire, England). Sections were stained with hematoxylin and then viewed and photographed using both light and dark field illumination on an Olympus BX-50 microscope (Olympus New Zealand Ltd., Lower Hutt, New Zealand). Non-specific hybridization was monitored by hybridizing at least two tissue sections from each age group (lamb and adult) with approximately equal concentrations of the sense RNA for each gene. Hybridization was considered to be specific when the intensity of silver grains, as measured by visual assessment, over a cellular type was greater than that observed in the area of the slide not containing tissue. For all genes, hybridization of the sense RNA over the tissue section was similar or lower in intensity to that observed on the areas of the slide not containing tissue of both the sense and antisense hybridized slides and thus was considered non-specific.

### Follicular classification

Classification of follicles was based on the system outlined by Lundy et al. [29]. Briefly, type 1/1a follicles consist of an oocyte surrounded by a single layer of flattened or mixed flattened and cuboidal cells. Type 2 follicles contain 1 < 2 layers of cuboidal granulosa cells whereas type 3 follicles contain 2 < 4 layers of cuboidal granulosa cells. Type 4 follicles have >4 layers of granulosa cells and a well defined theca but have not yet formed an antrum. Type 5 follicles have multiple layers of granulosa cells, a well defined theca and a defined antrum. All follicles with signs of degeneration (i.e. pyknotic granulosa cells, lack of a distinct basement membrane or degenerate oocytes) were excluded from the study. Ovarian sections from a minimum of eight animals, including at least three lambs and three adults, were examined for each gene studied. In addition, each follicle class was observed in a minimum of three animals. No differences in expression patterns between lamb and adults ovaries were noted in this study.

### Granulosa cell culture

Ovaries were collected from ewes following slaughter at the local abattoir, transported back to the laboratory at room temperature, washed in 3% bleach solution in PBS for 5 minutes, rinsed twice in PBS and stored in Leibovitz media containing 0.1% BSA, 100 U/ml penicillin and 100 μg/ml streptomycin. Follicles approximately 1–2 mm in diameter were dissected away from the ovaries and stored in Leibovitz media until collection of granulosa cells. The granulosa cells were collected by cutting follicles in half followed by manual scraping of cells from the follicular wall using a wire loop. Oocytes and follicular debris were removed from the cells using a micro-glass pipette. Remaining cells were collected by centrifugation at 300 g for 5 min at room temperature, washed once in 5 mls Leibovitz media, twice in 5 mls McCoys media (Sigma, Auckland, New Zealand) with 100 U/ml penicillin, 100 μg/ml streptomycin and 2 mM GlutaMAX-1 and resuspended using a syringe and needle. Cell viability was determined using trypan blue exclusion and 100,000 viable cells per well (250 μl total volume) were added in McCoys media containing 100 U/ml penicillin, 100 μg/ml streptomycin, 2 mM GlutaMAX-1, 5 ng/ml selenium (Sigma), 10 ng/ml insulin (Sigma), 5 μg/ml apo-transferrin (Sigma), 30 ng/ml androstenedione (Sigma), 3 ng/ml ovine FSH (purified in our laboratory; 1.4 X USDA-oFSH-19-SIAFP RP2), 1 ng/ml IGF-1 (Long-R3, GroPep, Adelaide, SA 5000, Australia) with varying doses (0–10 ng/ml) of purified human TGF-β1 and recombinant human TGF-β2 (R & D Systems, Minneapolis, MN). Cells were cultured at 37°C in a 5% CO2 incubator. Every 48 hours, 200 μl of media was removed from each well and replaced with 200 μl of warmed media that had been prepared at the start of the culture and stored at 4°C. Media samples from the last 48 hours of treatment were collected on day 6 of treatment and frozen at -20°C for later determination of progesterone concentrations by RIA. Unattached cells were removed by 2 washes with McCoys media at 37°C. Attached cells were lysed by incubating cells at 37°C in 100 μl distilled water for 1–2 hours followed by freezing at -70°C. All treatments were performed at least in triplicate with three independent pools of granulosa cells. Within an assay, individual values outside of 20% of the mean value for the treatment were discarded. Points in which at least 2 of the replicates were not within 20% of each other were regarded as missing data. This occurred for the 10 ng TGF-β1 measure of DNA in a single pool of granulosa cells.

### Measurement of DNA

The amount of DNA present in each well was determined by comparing binding of Hoechst 33258 dye (Sigma, final concentration in well of 10 μg/ml) in samples to calf thymus DNA standard measured with a Wallac 1420 plate reader at 350 nm for excitation and 460 nm for emission. Sensitivity of the assay (+ two SD of control buffer value) was 33 ng per well and the intra- and inter-assay co-efficients of variation (CV), based on variability of the 100, 250, 1000 and 2500 ng standard curve points were 3.9% and 8.8%, respectively. No samples were below the sensitivity of the assay.

### Measurement of Progesterone

Concentrations of progesterone in media were determined by RIA as described [30]. The sensitivity of the assay (90% maximum binding) was 13 pg/ml and the intra- and inter-assay CV, averaged for a standard pool sample at approximately 20%, 50% and 80% binding, was 8.3% and 19.7%, respectively. No samples were below the sensitivity of the assay.

### Determination of expression of TGFβRII mRNA in cultured granulosa cells

Granulosa cells were collected as described above and either frozen immediately after collection or plated in 6 well culture dishes at a density of 1.0–1.5 × 10^6 ^viable cells per well in 2 mls of control (i.e. no TGF-β) culture media described above for 48 hours. At this time, unattached cells were removed by washing the wells twice with PBS. RNA was collected using TRIzol according to the manufacturer's instructions. First strand cDNA was produced from total cellular RNA using the SuperScript™ preamplification system for first strand cDNA synthesis. An initial PCR was performed with 4 week old ovary RNA to obtain the ovine sequence of a region of the TGFβRII gene which spans introns 4 and 5 in the human sequence (AY675319) and a second set of primers was designed based on the ovine sequence (see table 1). Expression of TGFβRII was determined by PCR using the Qiagen Taq PCR core Kit (Biolab Scientific Limited) and the internal ovine primers listed in table 1 with the following conditions: initial denaturing cycle of 3 minutes at 94°C followed by 40 cycles of denaturing at 94°C for 1 minute, annealing at 55°C for 1 minute and extension at 72°C for 2 minutes and a final extension at 72°C for 10 minutes. cDNA generated from a 4 week old lamb ovary was run as a positive control whereas replacement of cDNA with water was used as a negative control. Expression of TGFβRII was assessed by visualization of DNA bands of the correct size following gel electrophoresis. Identity of product was confirmed by sequencing.

### Statistical analysis

Concentration of progesterone per μg DNA was calculated for individual wells before averaging for each treatment within each assay. Points in which at least 2 of the replicates were not within 30% of each other were regarded as missing data. Changes in the concentrations of progesterone in media and DNA content after culture were analysed with the general linear model procedure of SAS. Replicate was included in the model as baseline progesterone and DNA values varied among the granulosa cell pools. Differences between least square means were evaluated using least significant differences and were considered significant when p < 0.05. Data presented are least square means. The standard errors of least square means were 0.7 ng/well, 0.2 μg/well and 0.5 ng/μg for progesterone, DNA and p4 per DNA, respectively.

## Results

### *In situ *hybridization

#### TGF-β1

**Figure 1:**
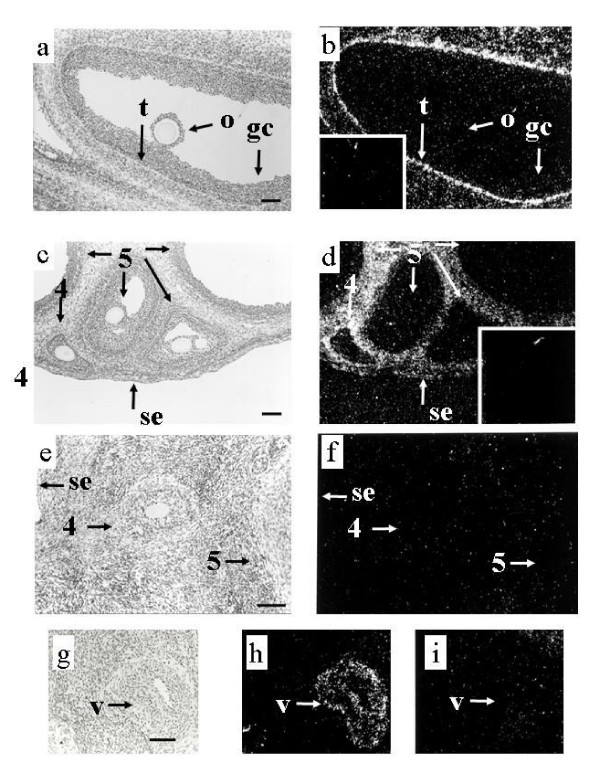
**Localization of expression of mRNA encoding TGF-βs in ovine ovaries. **Panels a and b contain corresponding light field and dark field views of a type 5 follicle from an adult ewe hybridized to TGF-β1 antisense RNA. Silver grains indicating hybridization of the TGF-β1 antisense RNA are observed concentrated in thecal (t) cells close to the basement membrane with no specific hybridization observed in either the granulosa cells (gc) or oocyte (o). The inset in panel b contains a dark field view of the same area of the tissue hybridized to the TGF-β1 sense RNA. Note the lack of specific concentration of silver grains over any cellular type. Panels c and d contain several type 5 (5) follicles and a type 4 (4) follicle in a 4 week old lamb hybridized to TGF-β2 antisense RNA. Note the lack of hybridization in the oocytes and granulosa cells of the type 4 and 5 follicles and the concentration of silver grains in thecal cells around the follicles as well as the stromal cells between the follicles and scattered cells of the surface epithelium (se). Observe the equal distribution of silver grains over the thecal cells. The inset in panel d contains a dark field view of the same area of the tissue hybridized to the TGF-β2 sense RNA. Note the lack of specific concentration of silver grains over any cellular type. Panels e and f contain corresponding light field and dark field views of an ovarian section obtained from an adult ewe hybridized to TGF-β3 antisense RNA. There is a lack of hybridization in the section including the granulosa and thecal cells of the types 4 (4) and 5 follicle (5) as well as the oocyte of the type 4 follicle, stroma tissue and the surface epithelium (SE). Panels g and h contain light field and dark field views of a blood vessel (v) from an adult ewe hybridized to TGF-β3 antisense RNA. Observe the specific hybridization in the wall of the vessel (v ). Panel i contains a dark field view of the same area of the tissue hybridized to TGF-β3 sense RNA. Note the lack of specific concentration of silver grains over any cellular type. Scale bar equals approximately 100 μm for all panels.

The mRNA for TGF-β1 was not observed in granulosa cells or oocytes of any follicles (Figure 1a,1b, table 2). However, TGF-β1 mRNA was observed in stromal and/or thecal cells of type 3 follicles, in the theca interna of type 4 and 5 follicles and also in the stroma and cells of the vascular system. Within the theca interna, the cells closest to the basement membrane usually had more intense signal than those further away (Figure 1a,1b).

**Table 2:** Summary of expression of mRNA encoding TGF-βs and receptors in ovine ovary.

Gene	Follicular type					Stroma	Vascular System
			
	1/1a	2	3	4	5		
TGFβ1	-	-	t	t	t	+	+
TGFβ2	-	-	t	t	t	+	+
TGFβ3	-	-	-	-	-	-	+
TGFβRI	o	o	o, gc, t	o, gc, t	o, gc, t	+	+
TGFβRII	-	-	t	t	t	+	+

+, expression observed; -, expression not observed; o, oocyte; gc, granulosa; t, theca

#### TGF-β2

The pattern of expression of mRNA encoding TGF-β2 was similar to that observed for TGF-β1, with hybridization limited to the thecal cells of type 3 and larger follicles (Figure 1c,1d, table 2). However, hybridization within the thecal layer appeared evenly distributed in contrast to the signal for TGF-β1 (compare panels a, b and c, d in Figure 1). Expression of TGF-β2 mRNA was also observed in some surface epithelium and stromal cells as well as cells associated with the vascular system.

#### TGF-β3

Expression of TGF-β3 mRNA was exclusive to cells associated with the vascular system of the ovary. Expression was not observed in the granulosa, theca, or oocyte of any follicle examined (Figure 1e,1f,1g,1h,1i, table 2).

#### TGFβRI

**Figure 2:**
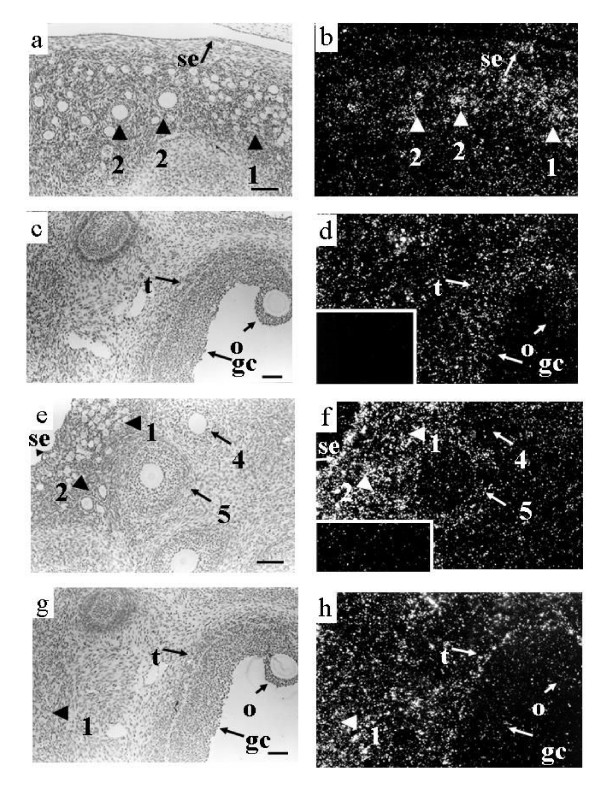
**Localization of expression of mRNA encoding TGF-β receptors in ovine ovaries. **Panels a and b contain corresponding light field and dark field views of several small follicles from a 4 week old lamb following hybridization to the TGFβRI antisense RNA. Note specific hybridization in the oocytes of types 1/1a follicles (1) and type 2 follicles. Observe that some cells of the surface epithelium also express TGFβRI. Panels c and d contain corresponding light field and dark field views of a type 5 follicle from a 4 week old lamb following hybridization to the TGFβRI antisense RNA. Note the hybridization signal in the granulosa (gc), theca (t) and oocyte (o) of the type 5 follicle. Signal was also observed in many stromal cells. The inset in panel d contains a dark field view of the same area of the tissue hybridized to TGFβRI sense RNA. Observe the lack of specific concentration of silver grains over any cellular type. Panels e and f contain corresponding light field and dark field views of several small follicles from a 4 week old lamb following hybridization to the TGFβRII antisense RNA. Note the lack of specific hybridization in the type 1/1a and 2 follicles. Expression was observed in the theca of type 4 and 5 follicles however, note the lack of expression in the granulosa cells and oocytes of these follicles. Note also that some cells of the surface epithelium also express TGFβRII. The insert in panel f contains a dark field view of the same area of the tissue hybridized to TGFβRII sense RNA. Note the lack of specific concentration of silver grains over any cellular type. Panels g and h contain corresponding light field and dark field views of a type 5 follicle as well as several type 1/1a follicles from a 4 week old lamb ovary hybridized to the TGFβRII antisense RNA. Note that hybridization is limited to the theca (t) of the type 5 follicle and several stromal cells and is not observed in the granulosa cells (gc) or oocyte (o) of the type 5 follicle. In addition, signal is observed in the stroma around the type 1/1a follicles (1) but is not observed in the type 1/1a follicles. Scale bar equals approximately 100 μm for all panels.

Expression of TGFβRI mRNA was observed in oocytes of all types of follicles (Figure 2a,2b,2c,2d, table 2). Granulosa and thecal cells of type 3 and larger follicles also expressed TGFβRI mRNA (Figure 2c,2d). Signal was also observed in the surface epithelium, stromal cells (Figure 2a,2b,2c,2d and luteal tissue (data not shown).

#### TGFβRII

Expression of TGFβRII mRNA was not observed in types 1,1a or 2 follicles (Figure 2e,2f,2g,2h, table 2). Also, in larger follicles TGFβRII mRNA was not detected in granulosa cells or oocytes (Figure 2e,2f,2g,2h). In type 3 and larger follicles, expression of TGFβRII was localized to the theca interna (Figure 2e,2f,2g,2h, table 2). As was observed with TGF-β1, expression of TGFβRII within the theca was most intense in the cells adjacent to the basement membrane (Figure 2e,2f,2g,2h). Signal was also observed in some cells of the surface epithelium (Figure 2e,2f), and in stroma (Figure 2e,2f,2g,2h) and luteal tissue (data not shown).

### Effects of TGF-βs on granulosa cell function *in vitro *and expression of TGFBRII in cultured cells

**Figure 3:**
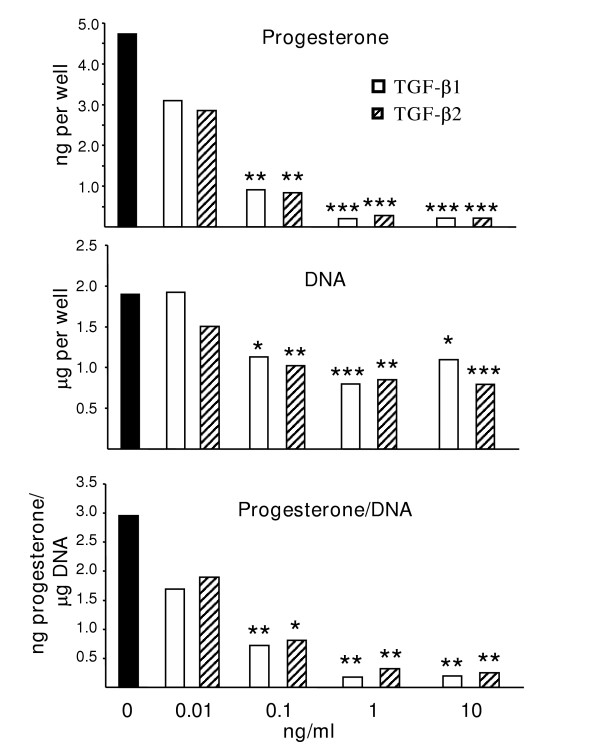
**Effects of TGFβs on granulosa cell function. **Effects of TGF-β1 and TGF-β2 on secretion of progesterone during the last 48 hours of culture (top), content of DNA at the termination of culture (middle) and progesterone concentration per μg of DNA. Values are expressed as the LS mean from 3 separate experiments. The dose of either TGF-β1 or TGF-β2 is indicated along the bottom of the graphs. For each variable, asterisk(s) indicates values that are different from the control (0) value (* p < 0.05; ** p < 0.01, *** p < 0.001). Comparisons were also made between the values obtained for TGF-β1 and TGF-β2 at each dose; however, no significant differences were observed at any dose tested.

**Figure 4:**
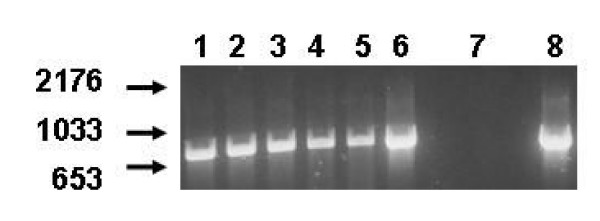
**Expression of TGFβRII in cultured granulosa cells. **Determination of expression of TGFβRII in granulosa cells immediately following collection and following 48 hours of culture. Lanes 1–3 contain PCR products (766 bases) following amplification with ovine TGFβRII primers from 3 separate pools of granulosa cells at the time of collection, lanes 4–6 contain PCR products (766 bases) following amplification with ovine TGFβRII primers from 3 separate pools of granulosa cells collected 48 hours after culture, lane 7 contains the negative control water blank whereas lane 8 contains the PCR product from the positive control 4 week old ovary sample. Migration of DNA molecular weight standards are indicated on the left hand side.

Both TGF-β1 and TGF-β2 inhibited progesterone synthesis of cultured granulosa cells, whether expressed as a function of number of cells placed in culture (Figure 3, top panel) or as a function of DNA content at the end of culture (Figure 3, bottom panel) with significant affects observed with as little as 0.1 ng/ml of either TGF-β. Treatment with either TGF-β also reduced DNA content at the termination of culture (Figure 3, middle panel). For both variables, no differences were observed between the effect of TGF-β1 and TGF-β2 at any dose of growth factor tested. In contrast to the lack of detectable expression of the TGFβRII mRNA observed *in situ*, freshly isolated or cultured granulosa cells expressed mRNA for the TGFβRII when assessed by RT-PCR (Figure 4).

## Discussion

In the ewe, expression of TGF-β1 and TGF-β2 mRNA in the follicle was limited to thecal cells during all stages of follicular growth examined. Furthermore, expression of TGF-β3 mRNA was not observed in any follicular cells. This is in contrast to the observed expression patterns for these proteins in other species where TGF-β1 and TGF-β2 have been localized to granulosa as well as thecal cells and sometimes also to the oocytes of many species [8,12,13,31-33]. Also in contrast to sheep, expression of TGF-β3 in cattle and cats was observed in the oocyte, theca and granulosa of follicles at various stages of development [12,13]. In pigs, the theca interna has been proposed to be the major source of TGF-β since granulosa cells express TGF-β1 mRNA without seeming to make the protein [11]. Similarly, expression of TGF-β2 mRNA has been observed in bovine oocytes, but no detectable TGF-β activity was observed [17], although other studies have demonstrated TGF-β protein in the oocytes using immunocytochemistry [12]. In addition, granulosa cells isolated from pigs and cattle produce little if any TGFβ bioactivity when cultured *in vitro *[9,11]. Thus, it seems likely that in some species, follicular TGF-β activity originates primarily from the thecal cells, with control of activity possibly occurring at several levels including gene transcription (this study), protein translation [11] or activation of the protein [17].

Similar to what has been observed in other species [32,34,35], expression of TGFβRI mRNA was observed in several different cell-types of the sheep ovary including the oocyte, granulosa cells, thecal cells, stroma, luteal cells and surface epithelium. While expression of TGFβRII mRNA was also observed in stroma, luteal cells and the surface epithelium, its expression within the follicle was limited to the theca. A similar pattern of expression for the TGFβRII mRNA was observed in mouse follicles, with expression most prominent in the theca and barely detectable in granulosa cells [8]. However, using immunocytochemistry, strong staining for TGFβRII has been observed in granulosa cells with no to little staining in oocytes and in the theca in other species [13,32,35-37]. The reasons for these observed differences in localization of the TGFβRII are uncertain but may be due to differences in techniques or species differences.

Expression of mRNA encoding all three TGF-β isoforms and the TGF-β type I and II receptors were observed in cells associated with blood vessels and both receptor types and TGF-β1 and 2 mRNAs were observed in the stroma surrounding follicles indicating a potential role for TGF-β in regulating certain functions in the ovarian stroma and vascular network. TGF-β is known to be important in regulating angiogenesis [38,39]. Moreover, in the ovary, both TGF-β1 and TGF-β3 mRNAs are upregulated during revascularization following autotransplantation of rat ovaries [40] further supporting a role for these factors in regulating vascular function.

The much more restricted pattern of expression of TGFβRII mRNA in sheep indicates that the TGFβRI may well be involved with other type II receptors in the signalling of other members of the transforming growth factor family. In agreement with this, TGFβRI has recently been shown to be involved in signalling of the oocyte-derived GDF-9 along with BMPRII [41,42]. In other species, GDF-9 has been shown to regulate granulosa cell mitosis and differentiation [6] and has been shown to be essential for normal follicular growth and development in both mice [43] and sheep [44,45]. Thus, expression of TGFβRI mRNA as well as BMPRII [46,47] in granulosa cells is probably mediating the effects of GDF-9. Localization of both of these receptors in granulosa cells from the type 3 (secondary) stage of development onwards is consistent with the presence of normal primary but not secondary follicles in both sheep [44] and mice [43] lacking biologically active GDF-9. Interestingly, in sheep, TGFβRI mRNA and BMPRII [46,47] are also both localized in oocytes from the type 1 (primordial) stage onwards suggesting that GDF-9 may also regulate oocyte function in this species.

The suppression of progesterone production and DNA content in granulosa cell cultures by TGF-β1 or TGF-β2 is similar to inhibitory to mild stimulatory effects observed in bovine, ovine and porcine granulosa cell cultures and contrary to the strong stimulatory effects observed in rodents [11,14-18,20-26]. The decreased DNA content observed following treatment accounts for some, but not all, of the decrease observed in progesterone concentration in the granulosa cell cultures. The suppression of progesterone synthesis indicates an anti-differentiative role for this growth factor as has been observed for other members of the TGF-β superfamily. The decreased content of DNA observed following culture could be related to a suppression of granulosa cell proliferation or survival. Since TGF-β can stimulate apoptotic pathways in concert with other factors [48,49], a role for TGF-β in regulating apoptosis of ovarian cells has been proposed.

No differences in the efficacy of TGF-β1 and TGF-β2 were observed in ovine granulosa cells. Similarly, TGF-β1 and TGF-β2 were equally efficacious in stimulating inhibin production in luteinized human granulosa cells [50] and in modulating gonadotrophin receptor expression in both rat and porcine granulosa cells [51]. Interestingly, while both TGF-β1 and TGF-β2 mRNA were synthesized by the theca interna, their spatial patterning within the theca was quite different. TGF-β1 mRNA was concentrated in the thecal cells closest to the basement membrane, similar to the localization observed for the TGFβRII mRNA. In contrast, TGF-β2 mRNA expression was observed throughout the thecal layer. The role, if any, of the apparent differential regulation of these two isoforms in subtypes of thecal cells is currently unknown.

Given the potent effects of both TGF-β1 and TGF-β2 on granulosa cell function *in vitro*, the lack of detectable expression of TGFβRII mRNA in these cells using *in situ *hybridization was very surprising. There are several potential explanations for these apparent conflicting results. It is possible that TGFβRII is expressed in ovine granulosa cells and the technique utilized simply failed to detect this message. The detection of mRNA encoding TGFβRII in isolated granulosa cells both before and after culture using RT-PCR would seem to support this assumption. However, it is possible that the isolation and culture of the granulosa cells potentially could be inducing expression of TGFβRII as most all cells in culture express TGFβRII [52]. In addition, strong expression of TGFβRII mRNA in luteal tissue is also consistent with up regulation of the TGFβRII in these cells as induction of progesterone synthesis by the ovine granulosa cells can be considered to indicate at least a partial luteinization of these cells. Finally, it is also possible that TGFβs are using another member of the type II receptor family to mediate their effects. The existence of a second type II receptor capable of mediating TGF-β effects is supported by the inability of cell lines expressing TGFβRII to bind to TGFβ2 but not TGFβ1 [53] and cell lines responsive to TGF-β without a detectible type II TGFβR [52].

## Conclusions

Expression of mRNAs encoding TGF-β1 and TGF-β2 as well as both type I and II TGF-β receptors were observed in the theca of small growing follicles indicating that TGF-βs may be regulating thecal cell function in an autocrine manner. Expression of mRNA encoding TGF-β type I and II receptors is also observed in luteal cells, stroma, the vascular system and surface epithelium suggesting that TGF-βs may also regulate other cell types in the sheep ovary. Since granulosa cells showed no evidence of expressing any of the TGF-β ligands and expression of the TGF-β type II receptor was equivocal, it seems likely that any TGF-β effects in granulosa cells in vivo are due to paracrine or endocrine actions and possibly regulated through an alternative type II receptor.

## Authors' contributions

AHB, LDQ and LJH cloned the ovine TGF-βs and receptors, completed sequencing projects and alignments, and performed the *in situ *hybridizations and PCRs. SL and KLR performed the granulosa cell bioassays including progesterone and DNA measurements. JLJ and KPM designed and co-ordinated the experiments, performed statistical analysis and drafted the manuscript. All authors read and approved the final manuscript.
